# Serum metabolomics signatures after an acute bout of combined traditional or high‐intensity tactical training in young adults

**DOI:** 10.14814/phy2.71094

**Published:** 2026-09-21

**Authors:** Zachary A. Graham, Khyatiben V. Pathak, Krystine Garcia‐Mansfield, Kaleen M. Lavin, Anakaren R. Torres, Jeremy S. McAdam, Timothy Broderick, Patrick Pirrotte, Marcas M. Bamman

**Affiliations:** ^1^ Healthspan, Resilience and Performance Florida Institute for Human and Machine Cognition Pensacola Florida USA; ^2^ Center for Exercise Medicine The University of Alabama at Birmingham Birmingham Alabama USA; ^3^ Department of Cell, Developmental and Integrative Biology The University of Alabama at Birmingham Birmingham Alabama USA; ^4^ Birmingham VA Health Care System Birmingham Alabama USA; ^5^ Early Detection and Prevention Division Translational Genomics Institute Phoenix Arizona USA; ^6^ Integrated Mass Spectrometry Shared Resource Translational Genomics Institute/City of Hope Comprehensive Cancer Center Duarte California USA; ^7^ Neurogenomics Division Translational Genomics Institute Phoenix Arizona USA

**Keywords:** combined training, exercise, high‐intensity, metabolomics

## Abstract

Alternative exercise prescriptions and doses would be expected to result in distinct signatures due to differences in duration and intensity. We tested two novel combined endurance and resistance exercise regimens to understand how differing prescriptions alter the timecourse of metabolomics response. Serum metabolomics for *n* = 37 untrained individuals was completed following traditional combined exercise [TRAD; *n* = 20 (11M/9F)] or high‐intensity tactical training [HITT; *n* = 17 (8M/9F)] before (pre), and immediately (h0), 3 and 24 h post‐exercise (h3 and h24, respectively). We found minimal metabolites had a group by time interaction (4 with FDR‐adjusted *q* values <0.10; 9 with nominal *p* < 0.01), but both stimuli resulted in large‐scale within‐group changes to the circulating metabolome. TRAD had greater numbers of differentially abundant metabolites (FDR <0.10) compared to HITT at h0 (451 vs. 358), h3 (446 vs. 348) and h24 (183 vs. 86). The metabolite classes altered were related to key energy substrates for both groups at h0 and energy replenishment for h3 and h24. In summary, our data describe the acute changes in the circulating metabolome following combined endurance and resistance exercise. Additionally, we show the two distinct doses of combined exercise led to generally similar patterns of responses.

## 1 INTRODUCTION

Exercise improves health through a coordinated physiological response in almost all tissue systems and cell types. Long term adaptation to exercise training prevents or slows the onset of many diseases and improves health and function in those with existing pathologies (Pedersen & Saltin, 2015). The diverse cues initiated by exercise are required to meet the demands of the activity while providing the molecular framework for protective long‐term changes in cellular architecture that build physiological reserve.

The metabolic demands of working tissue increase relative oxygen consumption by ~25‐fold during maximal exercise (Jones et al., 2021). Performing at this peak or at sustained submaximal levels requires elaborate inter‐ and intracellular communication. Increased accessibility to molecular profiling has greatly improved how we understand biological change at the molecular level (Sanford et al., 2020). Metabolomics in particular is experiencing rapid advancements as sensitivity and analyte coverage have improved resolution, allowing for discovery of novel metabolite‐specific mechanisms such as Lac‐Phe (Li et al., 2022). Thus, metabolomics allows further understanding of the molecular interplay required to maintain exercise performance, with unique signatures depending on mode and dose (Morville et al., 2020; Schranner et al., 2020).

Metabolomics has been performed across acute and chronic exercise studies (Jaguri et al., 2023), though most has been following acute endurance exercise (Schranner et al., 2020). US public health guidelines include both endurance and resistance training as integral to a healthy lifestyle (Piercy et al., 2018) and no timecourse study has investigated the acute circulating metabolomic response to combined endurance and resistance exercise. Recently, high‐intensity interval exercise has been adopted as an alternative to traditional steady‐state exercise, in particular in military personnel (Haddock et al., 2016). One of the major reasons for this increase in adoption is time, which is often a barrier to developing and maintaining a personal exercise program. With this backdrop, we recently completed a 12‐week, supervised, randomized controlled trial of two different combined training exercise prescriptions to test overall efficacy in key metrics of clinical performance and molecular adaptations (McAdam et al., 2025). The traditional combined training (TRAD) lasted ~90 min per session and our high‐intensity prescription, which we refer to as high‐intensity tactical training (HITT), lasted ~45 min. The general response of the training program was similar between the two groups but the acute multiomic responses were distinct (Lavin et al., 2023; Lavin et al., 2025). This study aimed to further describe the acute exercise response of the circulating metabolome in untrained individuals.

## 2 METHODS

### 2.1 Participants

Participants were recruited from the Birmingham, AL metro area as part of a larger parent trial (McAdam et al., 2025), with this cohort previously described (Lavin et al., 2023). Young, sedentary females and males 18–27 years of age completed a single acute bout of TRAD (*n* = 23; 13 M/9F, 22 ± 3 years) or HITT (*n* = 17; 9 M/8F, 22 ± 2 years). All individuals provided written, informed consent with protocol approval by the University of Alabama‐Birmingham Institutional Review Board (#F160512012). Screening and enrollment procedures have been described (McAdam et al., 2025). The study was completed in accordance with the Helsinki Declaration.

### 2.2 Exercise bout and blood collection

All participants completed four familiarization sessions before completing the acute exercise bout. Exercise was completed in the early morning after an overnight fast. Upon arrival, participants rested supine for 30 min with blood collected via venipuncture. Exercise started ~15 min post‐blood draw. HITT performed 10 rounds of a maximal intensity 30 s ‘On/Off’ program of box jumps, burpees, split squat jumps, kettlebell swings, 2 cycling sprints, a rowing sprint, battle ropes, wall balls, and dips. TRAD performed 30 min of cycle ergometry at 70% heart rate reserve (HRR) calculated from VO_2peak_. Both groups then performed whole‐body resistance training comprised of squats, knee extensions, heel raises, chest presses, overhead presses, seated rows, lat pulldowns, triceps pushdowns, biceps curls, and abdominal crunches. TRAD completed 3 × 13 reps at 13 rep max with ~60 s rest. HITT completed the same exercises in superset form at 3 × 9 reps at 9 rep max with 30 s rests between supersets. Exercise time was ~90 min for TRAD and ~45 min for HITT. Blood was collected immediately post‐exercise (h0), then participants consumed a protein drink (350 kcal; 11 g fat/16 g protein/47 g carbohydrate). Blood was collected 3 h (h3) and 24 h post‐exercise (h24). Participants were allowed to maintain their usual diet from h3 to h24 but were required to be overnight fasted for the h24 biospecimen collection. Blood was processed for serum by clotting at room temperature for 30 min, then centrifuging at 2000 *g* for 10 min at 4°C. Serum was aliquoted and frozen at −80°C. Sample sizes differ slightly compared to our previous report (Lavin et al., 2023) because of serum availability. TRAD: *n* = 20 at ‘pre’, *n* = 17 at ‘h0’, *n* = 16 at ‘h3’ and *n* = 18 at ‘h24’; HITT: *n* = 16 at ‘pre’, *n* = 14 at ‘h0’, *n* = 14 at ‘h3’ and *n* = 16 at ‘h24’.

### 2.3 Serum processing and mass spectrometry

Serum was the only biospecimen analyzed in this report. Serum metabolites were extracted using a 1:3 plasma: organic solvent (3:1 v/v methanol: acetonitrile) ratio. Solvent was spiked with four isotopically labeled internal standards (d8‐valine, ^13^C6‐phenylalanine, ^13^C6‐adipic acid, d4‐succinic acid), vortexed for 30 s, then incubated at −20°C for 10 min for protein precipitation. Supernatant metabolites were recovered by centrifugation at 15,000 *g* for 10 min at 4°C and subjected to untargeted metabolomics as described (Zhang et al., 2023). 10 μL/sample was pooled for QA/QC and was injected prior to sample batches for system conditioning and every five samples to monitor instrument performance, extraction efficiency, correct retention time drift and perform batch normalization (Dunn et al., 2011). Raw data were subjected to Compound Discoverer 3.2 for metabolite annotation and relative quantification (Najdekr et al., 2019).

### 2.4 Data processing and statistical analysis

Data were processed and visualized with R (v3.6) (R‐Core‐Team, 2014) using UpSet (Conway et al., 2017), pheatmap (Kolde, 2019) and mixOmics (Rohart et al., 2017) packages. Metabolite abundances were analyzed using *limma* (Law et al., 2020). Benjamini‐Hochberg false discovery rate (FDR) of <0.100 was the threshold. One participant failed outlier detection (6 > median absolute deviations; TRAD Male), and one participant was removed due to lack of ‘pre’ sample (HITT Female). The data matrix, metadata, and statistical outcomes are in File S1 (https://www.doi.org/10.6084/m9.figshare.25848985).

## 3 RESULTS

### 3.1 Participant characteristics

General participant descriptions were previously published (Lavin et al., 2023). The groups had no differences in age, height, weight, body composition, maximal aerobic (VO_2peak_) and anaerobic (Wingate) power, or strength.

### 3.2 Metabolites

**FIGURE 1:**
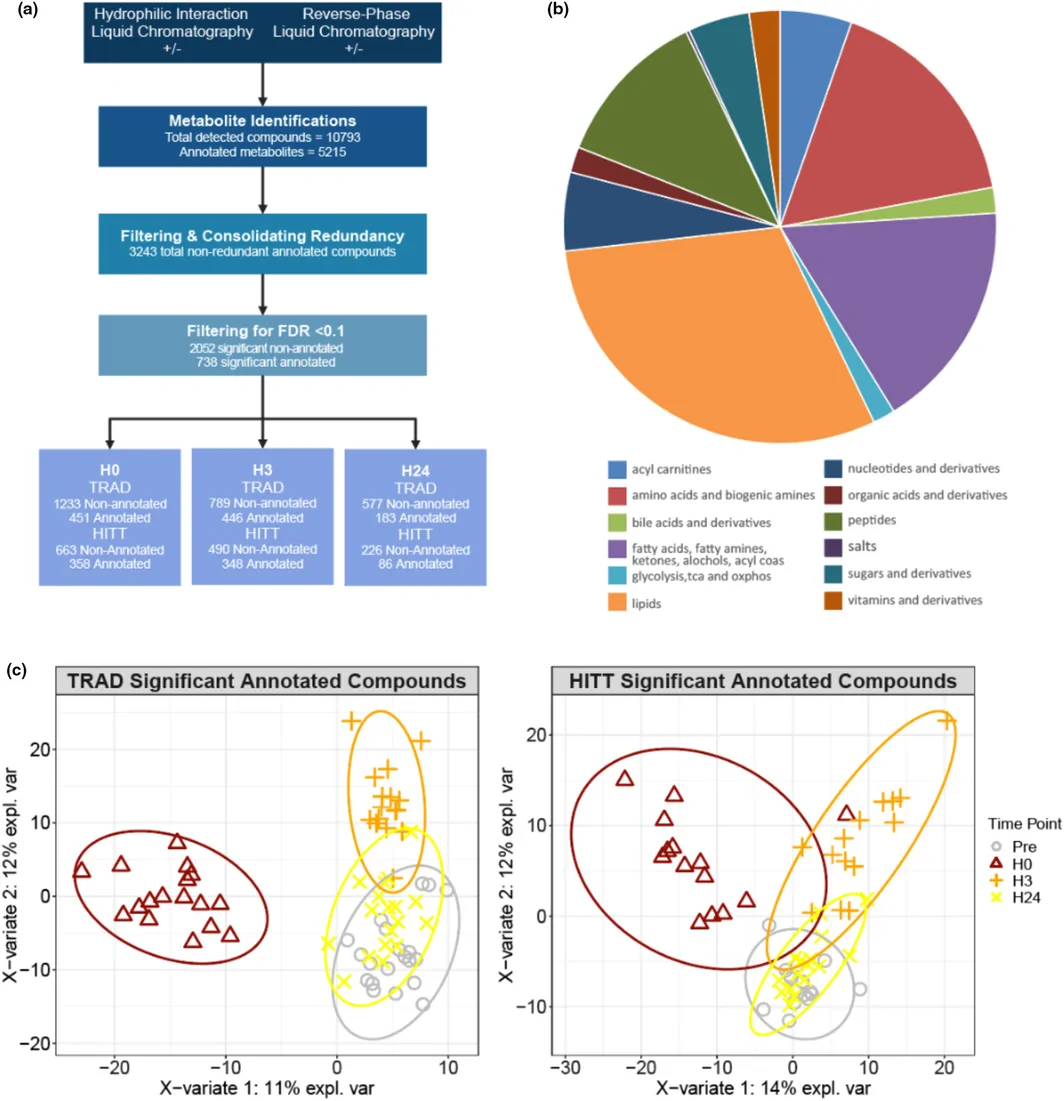
Serum metabolome identification. (a) Summary of metabolomics profiling including total compounds, annotated and unannotated compounds, metabolite verification and consolidating redundant metabolites. (b) Distribution of biochemical classes for 784 non‐redundant significant metabolites across comparisons (h0, h3, h24) between TRAD vs. HITT. (c) Partial least squares‐discriminant analysis (PLS‐DA) of differentially abundant metabolites (FDR‐adjusted *q* < 0.10) across time for TRAD (lower left panel) and HITT (lower right panel), respectively.

We detected 10,793 serum compounds across reverse phase and HILIC chromatographies in both positive and negative modes (Figure 1a). 5215 were annotated, with 3243 non‐redundant. 2052 total compounds were below FDR criteria in at least one comparison, including 738 annotated endogenous metabolites belonging to major metabolite classes (Figure 1b). PLS‐DA plots show unique clustering for both groups at h0 and h3 (Figure 1c) but not pre or h24.

The following analyses are on annotated metabolites only.

### 3.3 Between groups comparisons

**FIGURE 2:**
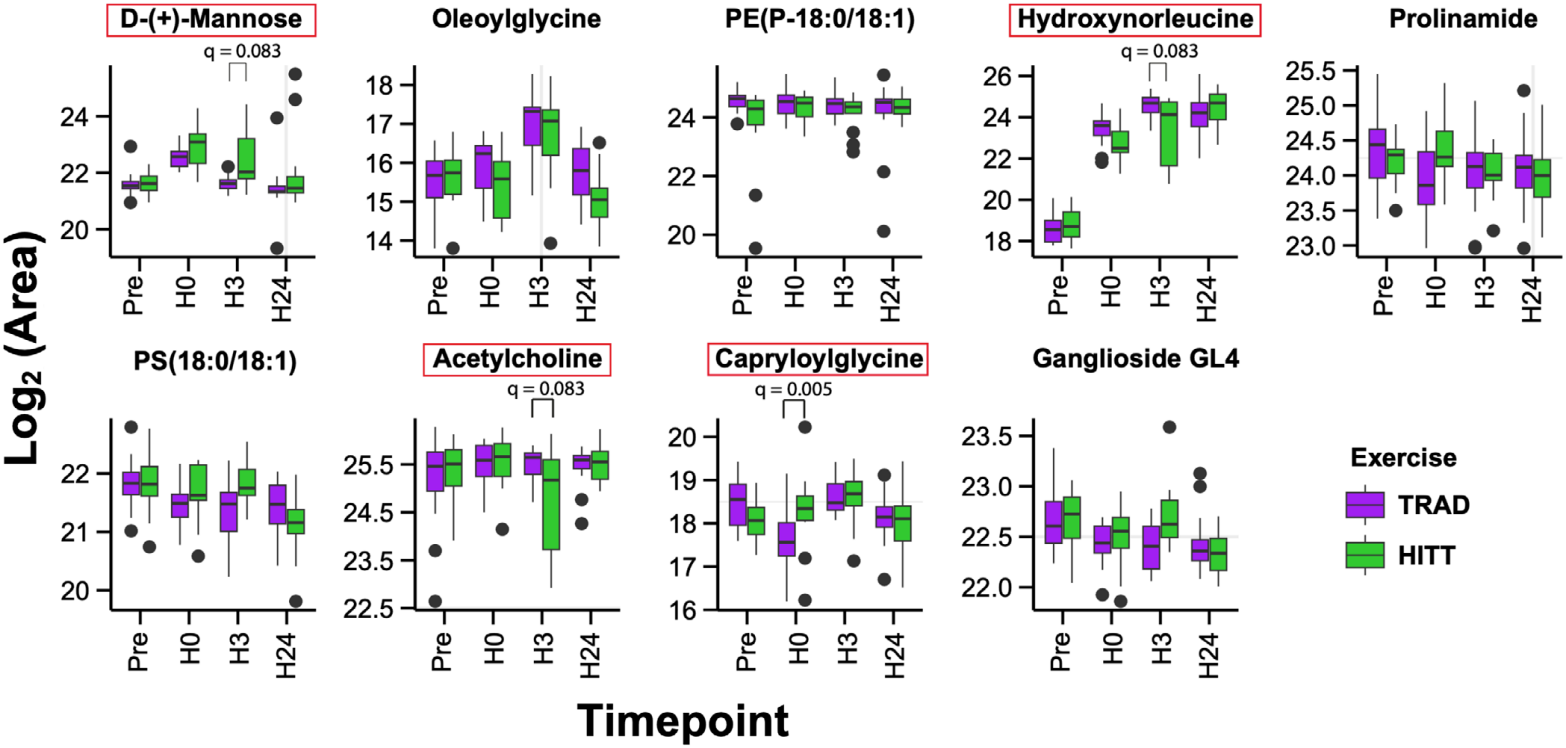
Differential circulating metabolomic responses for TRAD and HITT. Box and whisker plots log showing log‐transformed values for each metabolite per group and per timepoint. Metabolites highlighted with a red border are metabolites with a *limma‐*derived group difference at an FDR‐adjusted *q* < 0.10, with the timepoint with the difference shown with brackets and exact *q* value.

Comparisons of TRAD vs. HITT resulted in 9 differentially abundant serum metabolites with a nominal *p* value < 0.010 when comparing to baseline between the conditions (‘h0’: 2; ‘h3’: 5; ‘h24’: 2). Four of these metabolites had an FDR‐adjusted *q* < 0.10: capryloylglycine (*q* = 0.005, lower in TRAD at ‘h0’), hydroxynorleucine and acetylcholine (*q* = 0.083 for both, with both higher in TRAD at ‘h3’), and D‐(+)‐mannose (*q* = 0.083; lower in TRAD at ‘h3’). Figure 2c shows boxplots for each metabolite with *p* < 0.01 with red squares highlighting metabolites with *q* < 0.10.

### 3.4 Within‐group comparisons

**FIGURE 3:**
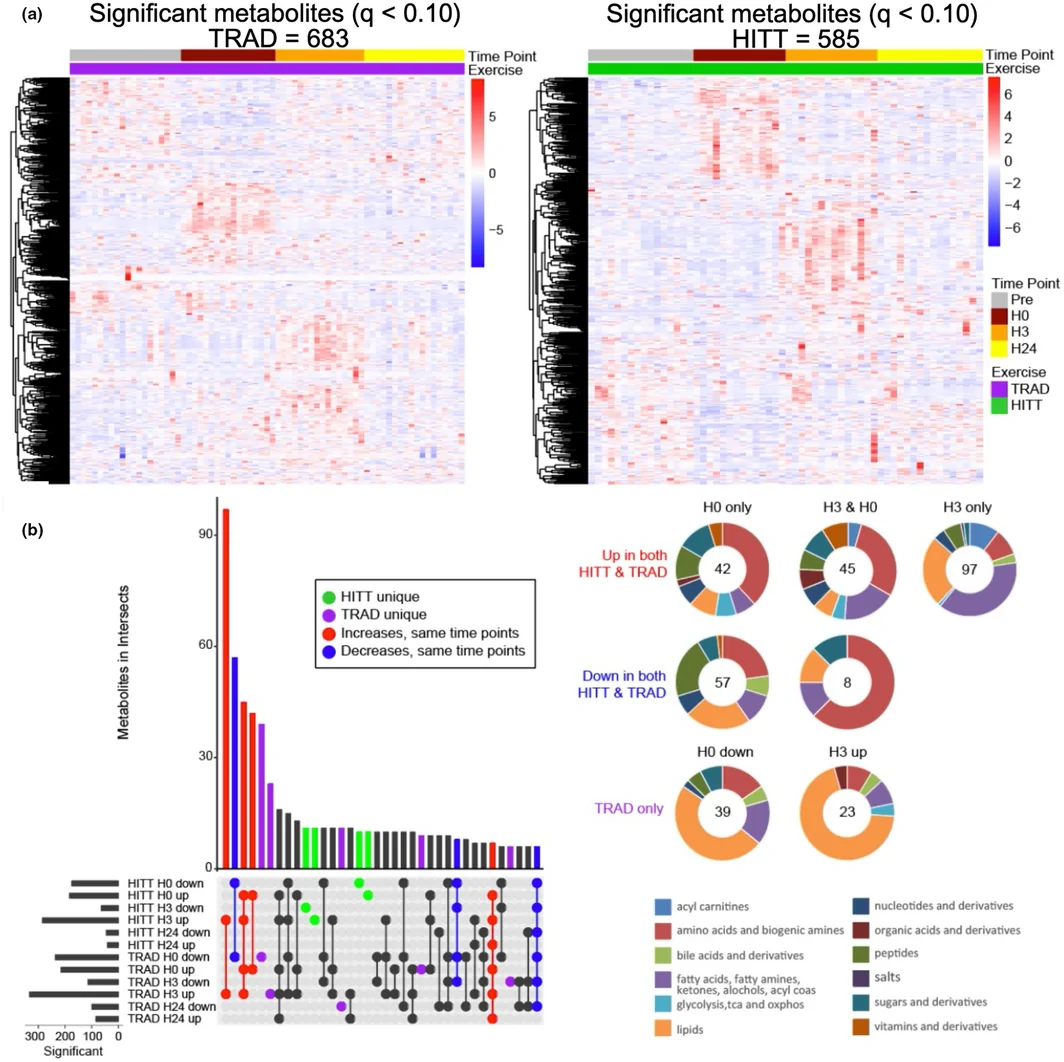
Within group comparisons across the exercise doses. (a) Hierarchical clustering of differentially abundant metabolites (FDR‐adjusted *q* < 0.10) for TRAD (Upper left panel) and HITT (Upper right panel). (b) Upset plot representing shared or unique metabolite signatures for TRAD and HITT alongside donut charts representing biochemical classes.

Almost all detected annotated serum metabolites showed a change in one of the exercise prescriptions at one or more postexercise timepoints: 683 and 585 metabolites were differentially abundant below FDR criteria in TRAD and HITT, respectively (Figure 3a). 451 metabolites (↑215, ↓236) were differentially abundant in TRAD at h0 compared to 358 (↑183, ↓175) for HITT. At h3, 446 metabolites (↑333, ↓113) and 348 metabolites (↑284, ↓64) were altered in TRAD and HITT, respectively. At h24, 183 (↑84, ↓99) metabolites were altered following TRAD and 86 (↑42, ↓46) following HITT.

Metabolite signatures and biological class membership are shown in Figure 3b, with a focus on the 6 intersections with >20 metabolites. The ‘h3 up’ profile had the most shared metabolites with 97 metabolites mostly related to lipid and fatty acid metabolism common to both groups. Both groups shared 57 downregulated metabolites at ‘h0’ and annotated to amino acids and biogenic amines, lipids, and peptides. ‘h0 up’ and ‘h3 up’ had 45 metabolites shared and were composed of biogenic amines, acyl carnitines, fatty acids and derivatives. A group of 39 metabolites was only decreased in TRAD at ‘h0’ and comprised phospholipids, fatty acids and derivatives, and amino acids. At h0 both groups shared 42 upregulated metabolites, mostly amino acids and biogenic amines. Lastly, a set of 23 metabolites was increased only in TRAD at ‘h3’ largely annotated to phospholipids.

### 3.5 Differential abundance patterns across time

**FIGURE 4:**
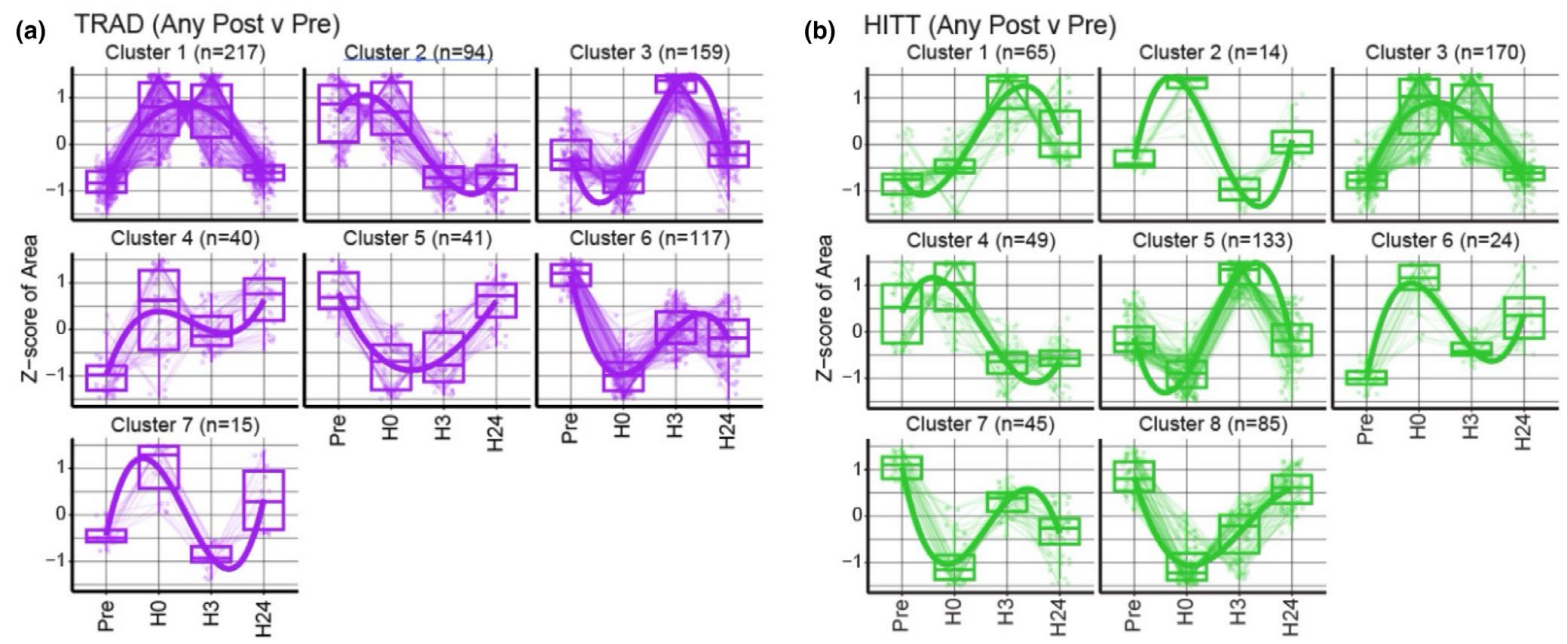
Unbiased clustering of all differential abundant metabolites compared to ‘pre’ for (a) TRAD and (b) HITT.

Unbiased clustering was used to determine patterns of change relative to ‘pre’ (cluster membership ≥10 metabolites; Figure 4a,b). Most serum metabolite signatures were shared between groups and are summarized in Table 1. There was a range of responses, from immediate increase (TRAD Cluster 1, HITT Cluster 3) or decrease (TRAD Cluster 5, HITT Cluster 8) with a return to baseline at h24, or immediate and sustained decrease (TRAD Cluster 6, HITT Cluster 7). Each exercise dose had a unique pattern. TRAD Cluster 5 did not share a signature with HITT, with h0 and h3 being equally downregulated, and it contained metabolites such as succinate. HITT Cluster 8 was also a distinct response with downregulation at h0 and continual upregulation at h3 and h24. This cluster contained features such as ornithine and glycine. All cluster memberships between the exercise groups can be found in File S2 (https://www.doi.org/10.6084/m9.figshare.32641431).

**TABLE 1:** Unbiased clustering of differentially abundant metabolites across time.

Any timepoint vs. ‘pre’					
Shared patterns		Shared between clusters	Shared % TRAD	Shared % HITT	Key metabolites
TRAD	HITT				
Cluster 1	Cluster 3	123	56%	72%	Glucose, aconitate, pyruvate
Cluster 2	Cluster 4	28	30%	57%	Cortisol, tryptophan, lactate
Cluster 3	Cluster 5	77	48%	58%	12,13 di‐HOME, palmitoylcarnitine, oleic acid
Cluster 4	Cluster 6	8	20%	33%	Glutathione
Cluster 5	n/a	n/a	n/a	n/a	Succinate, thymidine, asparagine
Cluster 6	Cluster 7	16	14%	36%	Phenylalanine, 1‐hydroxy‐L‐tryptophan
Cluster 7	Cluster 2	1	7%	7%	N8‐acetylspermidine
n/a	Cluster 8	n/a	n/a	n/a	Ornithine, glycine, citrulline

## 4 DISCUSSION

Moderate, continuous endurance exercise has been the predominant focus of metabolomics‐based acute exercise research (Schranner et al., 2020). While others have demonstrated the efficacy of subacute and chronic combined exercise training to change the resting metabolome (Jaguri et al., 2023), this is the first study to investigate how an acute bout of combined exercise alters the circulating metabolome and how different combinatorial exercise prescriptions impact this response.

We identified four metabolites that met FDR criteria between TRAD and HITT: capryloylglycine (lower in TRAD at h0), hydroxynorleucine (elevated in TRAD at h3), acetylcholine (lower in HITT at h3), and mannose (lower in TRAD at h3). Acetylcholine was unchanged across all timepoints in TRAD and was only down at h3 in HITT. High intensity exercise reduces circulating choline levels (Penry & Manore, 2008) and has been associated with reduced acetylcholine (Conlay et al., 1992). Choline was similarly reduced in both TRAD and HITT groups at h0 and h3 in our within group comparison, suggesting reduced choline availability was not a driver of the h3 reduction in HITT and another process, such as reduced acetyl CoA availability or lower choline acetyltransferase activity, may have had an effect. The difference in mannose levels at h3 could be indicative of the liver responding to glycolytic stress due to the sustained intensity and lower‐rest burden in HITT. Epinephrine results in elevation in circulating mannose derived mostly from the liver during glycogenolysis (Taguchi et al., 2005), and cellular uptake could be a potential energy source via hexokinase phosphorylation, conversion to fructose‐6‐phosphate, and then shuttled either upstream or towards glycogen or downstream towards pyruvate. Capryloylglycine is a medium‐chain fatty acid conjugate often used in cosmetic products. However, it has been described as a metabolite in mammalian systems. In pigs, capryloylglycine is a principal metabolite produced by the pancreas (Jang et al., 2019) and in humans it is elevated in skeletal muscle biopsies of older adults compared to younger adults (Wilkinson et al., 2020). Genetically‐induced mitochondrial β‐oxidation deficiencies lead to elevated capryloylglycine levels that are excreted in urine (Costa et al., 2000). Why capryloylglycine was reduced immediately after TRAD is unknown as glycine levels were similar across both groups, suggesting equal availability. It is possible levels of caprylic acid were lower but it was not among our annotated metabolites. Another possibility is TRAD reduces activity of glycine N‐acyltransferase, the enzyme that conjugates glycine to fatty acids. Any meaningful physiological information regarding hydroxynorleucine has not been established to our knowledge and it does not show up as an identified metabolite in the Human Metabolome Database, though norleucine does (Wishart et al., 2022). Very limited data exists for norleucine in itself and none in regard to exercise. Additional studies that repeat our exercise‐induced findings for capryloylglycine and hydroxynorleucine are needed, with rigorous mechanistic studies to better understand if they have relevant physiological roles post‐exercise.

TRAD had consistently more metabolites with differential abundance at each timepoint compared to HITT in our within‐group analyses. This is in line with our transcriptomics work from this same cohort that reported generally elevated numbers of differentially expressed RNA species in TRAD vs. HITT (e.g., long non‐coding RNA, protein coding RNA, microRNA) in serum‐derived extracellular vesicles and skeletal muscle (Lavin et al., 2023). This is likely due to a combination of factors such as the sustained metabolic demand from the continuous nature of the endurance portion as well as the greater length of exercise duration for the TRAD group (~90 min) compared to HITT (~45 min). Despite the ‘h0’ timepoint being a unique cluster as identified by PLS‐DA in both TRAD and HITT as shown in our PLS‐DA plot, the ‘h0’ and ‘h3’ timepoints had roughly equivalent numbers of metabolites for each group. There was a differential pattern as ‘h0’ had roughly identical numbers of upregulated and downregulated metabolites while ‘h3’ was composed largely of upregulated metabolites. The upregulated metabolites in ‘h3’ were mostly annotated to fatty acids, conjugated fatty amines, and ketones. These represented classes fall in‐line with systemic replenishment of energy stores that is supported by both TRAD and HITT having elevated levels of the adipose tissue‐released exerkine 12,13 di‐HOME, which increases fatty acid uptake in skeletal muscle (Stanford et al., 2018), elevated levels of palmitoylcarnitine, which is derived from palmitoyl‐CoA and shuttled across the inner membrane of the mitochondria (where it is subsequently converted back into palmitoyl‐CoA for β‐oxidation), and a host of other long chain fatty acids and their metabolites. The ‘h0’ timepoint was more associated with the active substrates of ATP generation (glucose, pyruvate, citrate, aconitate, fumarate) and the purine catabolism/salvage pathways (purine, guanine, hypoxanthine, uridine monophosphate), and is supported by unimodal studies using 45–60 min of moderate to moderate‐vigorous steady state cycling or traditional resistance training at similar post‐exercise timepoints (Morville et al., 2020; Thompson et al., 2007). Purine metabolism and ultimate salvage is more energy‐efficient than de novo formation to maintain ATP levels, but the elevation in the blood levels of these metabolites would be expected to be from working skeletal muscle. Xanthine oxidase, the enzyme that converts hypoxanthine to uric acid, is not expressed in skeletal muscle so these metabolites are likely taken up by the liver to be salvaged or further metabolized to uric acid (Miller et al., 2019).

Our combinatorial approach complicates direct comparisons against the literature as both TRAD and HITT were novel prescriptions. Our group has investigated the sex‐specific response in the metabolome from these participants, but that analysis did not include looking at the wholistic response to exercise (Lavin et al., 2025). However, the magnitude of response was similar to both acute resistance and endurance bouts of exercise that saw peak changes immediately, 15 min, and out to 3 h post‐exercise (Morville et al., 2020). In both groups, the resistance portion was completed last. Thus, there is potential that our metabolomic profiling would more mimic a resistance training prescription; however, the relative TRAD and HITT up and downregulated proportions at h0 and h3 timepoints do not align with resistance and endurance unimodal proportions at these same timepoints (Morville et al., 2020), suggesting our combinatorial intervention results in a unique metabolomic dynamic.

Our study has a few considerations. For sex as a biological variable, our randomization strategy included stratification by sex to ensure relative balance between HITT and TRAD and not necessarily sex‐specific comparisons. As such, we covaried for sex within our analyses to focus exclusively on the exercise response as we have reported the sex‐specific multiomic responses for these participants, as mentioned above (Lavin et al., 2025). Another important consideration is the supplemental protein drink after the ‘h0’ blood draw. This was used to mimic a real‐world exercise scenario and we cannot rule out that some of the shared metabolites observed to be changed at 3 h post‐exercise, or less likely, 24 h, between both groups were from the supplement. However, this concern is dampened by evidence showing our general patterns of change in classes such as lipids and amino acids match those from both endurance and resistance training acute response studies where participants remained fasted for serial blood analyses over 3 h post‐exercise (Morville et al., 2020). Lastly, our participants were untrained. It is likely that unaccustomed exercise in general was the key driver of the metabolomics response. Future studies utilizing trained individuals may allow for the ability to see exercise dose‐specific responses. In summary, our novel combined exercise prescriptions resulted in robust metabolomic changes across multiple timepoints post‐acute exercise that share major characteristics of unimodal exercise bouts, but also suggest distinct signatures related to exercise dose.

## AUTHOR CONTRIBUTIONS

**Zachary A. Graham:** Conceptualization; data curation; formal analysis; investigation; methodology; visualization. **Khyatiben V. Pathak:** Data curation; formal analysis; investigation; methodology; visualization. **Krystine Garcia‐Mansfield:** Formal analysis; investigation; visualization. **Kaleen M. Lavin:** Data curation; formal analysis. **Anakaren R. Torres:** Data curation. **Jeremy S. McAdam:** Data curation. **Timothy Broderick:** Funding acquisition; investigation; project administration; resources. **Patrick Pirrotte:** Resources; supervision. **Marcas M. Bamman:** Conceptualization; data curation; funding acquisition; investigation; methodology; project administration; resources; supervision.

## FUNDING INFORMATION

This study was funded by Department of Defense Office of Naval Research Grant N000141613159 (M.M.B., T.J.B.). Additional support was provided by the Department of Veterans Affairs Rehabilitation Research and Development Service Grant IK2RX002781 (ZAG) and the National Institutes of Health Grant P30CA033572 (PP). The views represented in this manuscript are not reflective of the United States Government or the Department of Veterans Affairs.

## CONFLICT OF INTEREST STATEMENT

No conflicts of interest.

## ETHICS STATEMENT

All individuals provided written, informed consent with protocol approval by the University of Alabama‐Birmingham Institutional Review Board (#F160512012) and were in accordance with the Helsinki Declaration. We have previously completed serum metabolomics analyses from this cohort focusing only on sex‐specific differences (Lavin et al., 2025) but all data and figures reported within this manuscript are novel with no overlap.

## Supporting information


File S1.

File S2.

## Data Availability

All raw data and relevant metadata are provided in Supplemental File S1.
